# Heart Failure in Elderly Patients: Medical Management, Therapies and Biomarkers

**DOI:** 10.3390/ph18010032

**Published:** 2024-12-30

**Authors:** Paulina Nadziakiewicz, Wioletta Szczurek-Wasilewicz, Bożena Szyguła-Jurkiewicz

**Affiliations:** 1 Student’s Scientific Society, 3rd Department of Cardiology, Faculty of Medical Sciences in Zabrze, Medical University of Silesia, 40-055 Katowice, Poland; s81184@365.sum.edu.pl; 2 Department of Pharmacology, Faculty of Medicine, University of Opole, 45-052 Opole, Poland; 3 2nd Department of Cardiology and Angiology, Silesian Center for Heart Diseases, 41-800 Zabrze, Poland; 4 3rd Department of Cardiology, Faculty of Medical Sciences in Zabrze, Medical University of Silesia, 40-055 Katowice, Poland; bjurkiewicz@sum.edu.pl

**Keywords:** heart failure, biomarkers, elderly patients, pharmacological treatment, comorbidities

## Abstract

Heart failure (HF) is a common condition and one of the main morbidity and mortality factors in elderly patients. The incidence of HF progressively increases with age, reaching >10% in those aged 70 years or over. In the elderly population, both the diagnosis and the management of HF prove challenging, often requiring specialized care and a multidisciplinary approach. In seniors, atypical presentation of HF is much more common than in younger patients; thus, a holistic assessment with biomarkers related to HF allows for early diagnosis and accurate risk stratification in this group of patients. This article reviews the clinical and diagnostic differences in elderly patients with HF, highlighting the presence of comorbidities, frailty, cognitive impairment, and polypharmacy, as well as discussing potential biomarkers that may have clinical application in this population.

## 1. Introduction

Heart failure (HF) is a major public health problem, generating high healthcare-related costs. It can be defined as a clinical syndrome associated with cardiac abnormalities affecting structure and/or function. In the natural course of the condition, reduced cardiac output can be observed with elevated intracardiac filling pressures [1]. The prevalence of HF is strongly associated with age, increasing from around 1% in patients aged <55 years up to >10% in elderly aged over 70 years [2]. The number of elderly patients with HF is steadily growing due to better medical care and increasing life expectancy.

Elderly patients with HF are a varied group, including both patients with reduced, mildly reduced, and preserved ejection fractions (HFrEF: HF with reduced ejection fraction, HFpEF: HF with preserved ejection fraction, HFmrEF: HF with mildly reduced ejection fraction). Study shows that in patients over 60 years old, diastolic left ventricular dysfunction is more common, with a median prevalence of 36.0% (range 15.8–52.8%), while systolic dysfunction has a median of 5.5% (range 3.3–9.2%) in this group [2]. Thus, elderly patients with HF constitute an etiologically and functionally heterogeneous group of patients that requires an interdisciplinary approach due to comorbidities, which may additionally complicate the course of the HF. Furthermore, the clinical course of this condition may often be difficult to predict in the elderly population. This article reviews the clinical and diagnostic differences in elderly patients with chronic HF (CHF), highlighting the presence of comorbidities, frailty, cognitive impairment, and polypharmacy, as well as discussing potential biomarkers that may have clinical applications in this population.

## 2. Heart Failure in Elderly

In the group of patients with CHF aged over 65 years old, the overall course of the condition varies from other age groups. In older patients, the percentage of women affected is higher and the ratio of genders is more balanced [3]. Moreover, they usually suffer from diastolic HF. In the younger age group, however, most patients suffer from systolic HF [1], and men are affected more frequently. The most common causes of HF overall are coronary artery disease and hypertension. Opposed to the general population, HF etiology in the elderly is more likely to be related to valvular heart disease (VHD), with the most common VHD affecting the elderly being aortic stenosis. Other factors that contribute to the development of HF can also be observed in the elderly, like left ventricular hypertrophy and diastolic dysfunction [4]. HF characteristics vary across the elderly population. Patients aged 65 to 74 years more frequently had lower left ventricular ejection fraction (LVEF), with an ischemic etiology of HF. Before and during index hospitalization, they oftentimes required percutaneous coronary intervention or coronary artery bypass grafting. In turn, patients aged over 75 years old had a lower percentage of ischemic etiology and a higher average LVEF when compared to the other group [5]. Those patients may also vary regarding the prevalence of HFrEF and HFpEF. HFrEF was more common in men, while HFpEF occurred more frequently in women. Research also showed a declining trend of HFrEF incidence in the elderly [4]. When it comes to prognosis, among elderly patients hospitalized with HF, median survival is approximately 2.5 years, with 25% of patients dying within 1 year. While for all types of HF patients indiscriminately, 1-year and 5-year mortality rates after diagnosis were 20% and 53%, respectively [1]. The mortality rate in patients aged over 65 was greater than in younger patients, and the prognosis was significantly worse for patients over 75 [5].

Diagnosis of HF in the elderly is challenging, as the condition may have an unusual presentation in this group. Patients instead of typical symptoms that include dyspnea, fatigue, ankle swelling, and edema present with atypical, nonspecific symptoms distorting the clinical manifestation of the disease. These symptoms include tiredness, altered mental status, depression, and loss of appetite [6]. In the study conducted in a geriatric outpatient clinic in the Netherlands on 206 patients, the diagnosis could be confirmed in only 46% of geriatric patients with suspected HF. Only one-third of the patients with HF presented with typical symptoms [6].

Elderly patients are more likely to have multiple comorbidities, which play an important role in the treatment, prognosis, and diagnosis of HF. In the Interdisziplinäres Netzwerk Herzinsuffizienz registry, seven or more comorbidities were present in approximately 50% of the patients [3]. Another study investigating patients aged over 65 years old found that around 90% of participants had at least 3 comorbidities, and over 60% had at least 5 [7]. Furthermore, patients with a higher number of comorbidities were at a higher risk for all-cause mortality in patients with HF [8]. The Charlson Comorbidity Index (CCI) is the most extensively studied comorbidity index validated to assess the presence and degree of comorbidity in the elderly, currently used in geriatric practice [9]. It has been reported to be reliable and to provide a good correlation with mortality and survival outcomes, and it accounts for the effect of age [10]. However, studies provide conflicting evidence for CCI index in elderly patients with CHF. The ‘Osservatorio Geriatrico Regione Campania’ study finds that CCI does not predict long-term mortality in elderly subjects with CHF [11]. Another study states that high CCI, along with reduced EF and poor physical functionality, was associated with high mortality [12].

Atrial fibrillation (AF) is one of the most common comorbidities coexisting with HF. The proportion of patients with HF who develop AF increases with age and HF severity, making it especially prevalent in the elderly. Studies conducted on the Polish population show that nearly 40% of patients with HF also have AF [13,14]. Patients with AF have higher short- and long-term mortality after hospital discharge and a higher risk of readmission. The mean age of studied patients with both AF and HF was 72 (62–79) [5]. In a group of patients aged over 75 years old, AF often leads to stroke, which has a higher incidence in this group. Moreover, those patients are frequently undertreated with anticoagulants because of a higher risk of bleeding [13].

Another important comorbidity in elderly patients with HF is diabetes mellitus type 2 (DM-2). DM-2 and HF, due to their pathophysiology, are inclined to coexist, with each disease independently increasing the risk for the other. In HF cohorts, including patients with HFrEF and HFpEF, the co-occurrence of DM-2 ranges from 10% to 47% in different studies. The pathophysiological mechanism of this incidence is based on the acceleration of atherosclerosis via vascular smooth muscle cell proliferation and inflammation that is directly connected with hyperglycemia and hyperinsulinemia [15]. DM-2 can also cause cardiovascular autonomic neuropathy, which has potentially life-threatening complications including arrhythmias and silent myocardial ischemia [16].

Chronic kidney disease (CKD) is highly prevalent in the elderly, with renal dysfunction affecting as many as 60% of HF patients ≥ 75 years [17]. CKD and HF have a bidirectional interaction; CKD may worsen cardiovascular function, causing hypertension and vascular calcification, while HF may worsen renal function through the effects of neurohormonal and inflammatory activation, increased venous pressure, and hypoperfusion. Furthermore, CKD has been consistently associated with worse HF prognosis and is commonly recognized as a major independent determinant of increased mortality and morbidity in HF [1]. When it comes to the elderly with chronic HF and CKD, the one-year prognosis is different depending on the presence of frailty. Patients with CKD present a worse clinical profile, with more advanced HF, comorbidities, and frailty. However, CKD was found to be associated with a worse prognosis only in non-frail patients [17].

## 3. Frailty Syndrome in Elderly Patients

Frailty is a clinical syndrome associated with age and characterized by a decrease in physiological reserve under stressful conditions, leading to a state of vulnerability that entails an increased likelihood of adverse events [18]. Frailty has over 70% prevalence in patients aged over 70 years old hospitalized for HF [19]. Furthermore, frail patients affected with CHF have an overall increased risk of hospitalization and mortality. Studies also suggest that stratifying patients with HF based on frailty status offers valuable prognostic insights and can help guide priorities for HF interventions and management [20]. Thus, identifying and accurately characterizing frailty status in patients with HF is crucial in order to minimize the significant negative impact of frailty on CHF. Geriatric assessment can be useful to identify the main factors of frailty from the outset. There are multiple scales for frailty assessment, and the major ones [frailty index by Rockwood and Mitnitski, Fried phenotype from the Cardiovascular Health Study, FRAIL (Fatigue, Resistance, Ambulation, Illnesses, & Loss of Weight) scale, and the Hubbard modified frailty score] have comparable accuracy in predicting death and physical limitation in geriatric patients [21]. Cardiovascular rehabilitation plays an important role in evaluating and treating frailty. Its proven beneficial effects on muscle mass and strength, as well as mobility, habitual physical activity, social interaction, cognitive performance, mood, and vitality, were significant enough to be regarded as one of the best comprehensive therapies available in clinical practice with the potential to mitigate frailty and its downstream effects [22].

## 4. Pharmacotherapy in Elderly

Elderly patients are underrepresented in most trials investigating drug treatment for HF. Therefore, the recommendations for treatment in this group are more or less based on subgroup analysis and expert opinions. However, the majority of trials have not demonstrated age-dependent heterogeneity in the efficacy or safety of medical treatment for HF [23]. The pre-specified subgroup analyses in the major clinical trials investigating angiotensin-converting enzyme inhibitors (ACEI) [24], beta-blockers (BB) [25], mineralocorticoid receptor antagonists (MRA) [26], and sodium-glucose cotrasporter2 inhibitors (SGLT2i) [27,28] show that in patients with HFrEF, both in elderly and other age groups, the therapy yields similar results. Thus, pharmacotherapy in the elderly is recommended to follow the guidelines established for other age groups.

The current treatment regimen assumes the fastest possible initiation and rapid escalation of ACEI, BB, and MRA dosages simultaneously with the initial optimal dose of SGLT2i [1,29]. Simultaneous initiation takes place at the initial (low) doses recommended for HFrEF (except for SGLT2i, which are dosed from the beginning at the optimal dose) [1,29]. Especially in older patients, monitoring of potency and side effects (including kidney function) is of great importance in establishing the optimal regimen for each individual.

Out of the four main groups of drugs used in HF treatment, ACEIs were the first class of drugs shown to reduce mortality and morbidity in patients with HFrEF. They are recommended in all patients unless contraindicated or not tolerated. However, the 2021 guidelines strengthen the indication for angiotensin receptor-nephrilysin inhibitor (ARNI) as a replacement in ambulatory patients with HFrEF who remain symptomatic despite optimal treatment with ACEI/angiotensin receptor blockers (ARB) [1]. It is important to note that no randomized controlled studies that focused on elderly patients with ACEI or ARB were conducted. A meta-analysis of five randomized controlled trials with ACEI in patients with ischemic etiology of HF or left ventricular systolic dysfunction reported that there was a nonsignificant age-by-treatment interaction for mortality, reinfraction, and rehospitalization [30]. Few observational studies were conducted on this topic. An older review of multiple observational studies concurs with the results from the sub-analysis, validating the effectiveness of ACEI in the elderly [31]. Another observational study on elderly people with HFrEF and CKD concluded that treatment with ACEI/ARB was associated with a lower rate of cardiovascular events [32]. When it comes to angiotensin receptor-neprilysin inhibitors (ARNI), a subgroup analysis of the PARADIGM—HF (Prospective Comparison of ARNI With ACEI to Determine Impact on Global Mortality and Morbidity in Heart Failure) trial demonstrated consistent risk reduction in the primary endpoint of cardiovascular mortality or hospitalization for HF regardless of age. The trial enrolled almost 20% of patients aged ≥75 years, including 7.0% aged ≥80 years and 1.4% aged ≥85 years. It found that even in very elderly patients, the effect of ARNI on heart failure hospitalization seemed qualitatively and quantitatively similar to its effect in younger patients [33].

BBs play a major role in HF treatment. Controlled prospective clinical studies documented the efficacy of bisoprolol, carvedilol (and prolonged-release metoprolol succinate, as included in both the European and American guidelines [1,29]. Another beta-blockerused in HF is nebivolol; similarly to carvedilol, it has a vasodilatory effect. Interestingly, nebivolol is included as an evidence-based beta-blocker in the ESC guidelines but not the CCS or AHA/ACC/HFSA guidelines [1,29]. All of those drugs are eligible for treatment in elderly patients, as stated in the following trials. In the SENIORS (Study of Effects of Nebivolol Intervention on Outcomes and Rehospitalization in Seniors with Heart Failure) trial, therapy with nebivolol was compared with placebo in patients aged ≥70 years. Therapy with nebivolol led to a significant reduction of the primary endpoint all-cause mortality and cardiovascular hospitalizations regardless of the ejection fraction value [34]. A similar trial, including a considerable percentage of elderly patients, was conducted with metoprolol succinate, comparing its effect with placebo. A retrospective subgroup analysis found a comparable reduction regarding mortality and morbidity in patients aged over 69 and younger participants [35]. The CIBIS-ELD (Cardiac Insufficiency Bisoprolol Study in Elderly) trial was a superiority trial comparing therapy with the beta-blockers bisoprolol and carvedilol in older patients. It found no difference regarding tolerance or achieved target dose between the two [36]. However, patients with bisoprolol more often suffered from bradycardia, whereas carvedilol led to a reduction in the forced expiratory volume [36]. It shows the importance of individual approach to pharmacotherapy, as the choice of suitable BB should be tailored to patients’ needs and comorbidities.

Since the release of 2021 ESC guidelines, MRA (eplerenone and spironolactone) are recommended in all patients with HFrEF. Their use is associated with a reduction in HF symptoms, the risk of hospitalization for HF, and mortality. MRAs block receptors that bind aldosterone and other steroid hormones (e.g., corticosteroid and androgen) receptors, with different degrees of affinity. Eplerenone is more selective to the aldosterone receptor, thus causing less gynecomastia than spironolacton [1]. Similarly to ACEI, their impact on the elderly is based on prespecified subgroup analyses. Both in the RALES (The Randomized Aldactone Evaluation Study) and in the EMPHASIS-HF (Eplerenone in Mild Patients Hospitalization and Survival Study in Heart Failure), the analyses concluded that older HF patients benefit from treatment with an MRA to a similar extent as younger patients [37,38]. In a meta-analysis including 1756 patients ≥ 75 years old (352 from the RALES trial, 657 from the EMPHASIS trial, and 747 from the TOPCAT (Treatment of Preserved Cardiac Function Heart Failure With an Aldosterone Antagonist trial), MRAs were associated with lower cardiovascular mortality, morbidity, and hospitalization. The beneficial effect observed was higher in elderly patients with reduced LVEF [39]. The most important adverse effect of MRAs treatment is hyperkaliemia, which is more common in the elderly, especially with concomitant medication with an ACEIs or an ARBs; thus, in this group, renal function and electrolytes should be controlled regularly.

SGLT2i were introduced to the pharmacotherapy of HF fairly recently. They were initially observed to reduce the HF hospitalization rate in diabetic patients and, after multiple trials, became an important pharmacological group in HF treatment even in patients without DM-2 [1]. The current guidelines [1,29] strongly recommend (class I) dapagliflozin or empagliflozin in patients with HF (New York Heart Association class II–IV) with reduced left ventricular ejection fraction (LVEF ≤ 40%) to reduce the risk of hospitalization for HF and death. Elderly patients were widely represented in SGLT2i clinical trials, and sub-analysis has confirmed the benefit of these drugs in this specific population [27,28].

Diuretics are considered the basis of symptomatic HF treatment. In everyday clinical practice, they are used in acute HF treatment for patients with volume overload and in patients with compensated HF to maintain a stable state [1]. Especially loop diuretics frequently are the only drugs that can adequately control fluid retention in HF. Whereas thiazide diuretics may be considered in patients with hypertension, HF, and mild fluid retention, loop diuretics are the preferred diuretic agents [29]. However, the quality of the evidence regarding diuretics is lacking, and their effects on morbidity and mortality have not been extensively studied in randomized controlled trials [1]. Loop diuretics have been studied among older patients hospitalized for decompensated HF who were not taking diuretics before hospitalization. It concluded that loop diuretic prescription at discharge is associated with a significantly lower risk of 30-day all-cause mortality and HF readmission [40]. Loop diuretics were not extensively studied in CHF in elderly patients.

Despite the proven benefits of guidelines-based pharmacotherapy and the reassuring safety profile of most drugs, the issue of undertreatment in the elderly population still prevails. In the Euro Heart Failure Survey II, underuse and underdosage of medications recommended for HF were common in patients over 80 years old with HFrEF [41]. Other studies report similar results showing that drugs such as BBs and ACEIs are less prescribed in eligible patients over 75 years of age [42,43]. Therapy management may prove difficult in this group, as elderly individuals have lower tolerance and a greater propensity for developing adverse drug reactions. The homoeostatic systems of elderly subjects are very fragile, and the management of HF accompanied by numerous comorbidities requires a holistic approach toward the patient. It is prudent to start HF pharmacotherapy in older patients at lower doses, then slowly and carefully up-titrate to target doses to prevent intolerance and adverse drug reactions while providing adequate treatment [44].

Due to the complexity of pharmacotherapy, patients with HF are at higher risk of adverse reactions. Furthermore, there are a number of commonly used medications that are relatively or absolutely contraindicated in patients with HF because they can cause exacerbations of HF or harmful interactions with the administered drugs [45]. That makes drug-induced exacerbation or decompensation of established HF a relatively common occurrence. It is important to acknowledge the usual mechanisms by which drugs can exacerbate HF, which are sodium retention, negative inotropic effect, and direct cardiotoxicity. Nonsteroidal anti-inflammatory drugs deserve special attention, as they are one of the most common groups taken by patients worldwide. They are relatively contraindicated as they may precipitate acute decompensation in patients with HF [46]. As some patients with HF may also suffer from angina, it is important to note that verapamil and diltiazem increase HF-related events in patients with HFrEF and therefore should be substituted by other safer alternatives such as ivabradine and trimetazidine [1,47]. In the treatment of hypertension, aside from non-dihydropyridine calcium channel blockers (diltiazem and verapamil), centrally acting agents, such as moxonidine, are contraindicated as they are associated with worse outcomes [48]. On the other hand, alpha-blockers according to ESC guidelines have no effects on survival and are therefore not indicated, while 2022 AHA guidelines indicate that they may cause or exacerbate HF and should be avoided [1,29]. In cases of diabetes and HF coincidence, treatment should avoid thiazolidinediones (glitazones), as they cause sodium and water retention and an increased risk of worsening HF and hospitalization [49]. Also, sulfonylureas were associated with a higher risk of HF events in some analyses [1].

## 5. Polypharmacy and Its Impact on Elderly

In HF, the gradual incorporation of multiple medications and a complex treatment regimen is common and recommended by ESC guidelines for the treatment of acute and chronic HF [50]. Adequate pharmacotherapy is also crucial to controlling comorbidities, lowering mortality, and potentially improving the quality of life. In the elderly, where the number of comorbidities is usually greater, each could require additional specific treatment, thus further increasing the number of drugs prescribed. Study shows that the vast majority (84% at admission and 95% at discharge) of older patients take at least 5 different medications, and nearly half of the investigated population (42% at admission and 55%) at discharge took over 10 different ones [51]. Elderly patients are highly susceptible to the adverse effects of polypharmacy due to age-related transformation in pharmacokinetics and pharmacodynamics [52] and coexisting conditions like frailty and mental impairment.

Among the drugs used in elderly patients with HF, warfarin could be especially harmful in polypharmacy due to its multiple potential interactions [53]. The last guidelines for atrial fibrillation [54] recommend direct oral anticoagulants as a first-line drugs, but many elderly patients are still taking warfarin due to its high price point [55]. Furthermore, there are also some conditions that still require vitamin K antagonists usage. Patients with mechanical valves or left ventricular assist devices are treated with vitamin K antagonists in long-term oral anticoagulation therapy. Both patients with those conditions or atrial fibrillation make up for a significant number of elderly patients still taking warfarin, therefore at higher risk of adverse drug reactions [56]. Unfortunately, in Poland, almost 50% of optimally treated elderly HF patients were taking additional medications and supplements for self-treatment, such as analgetics, herbal products, and mineral products, further increasing the probability of interactions [57]. A similar situation was observed in the USA, where more than half of the HF patients used at least one dietary supplement [58].

Another important issue is adherence to therapy. A study by Giardini et al. shows that adherence is usually low in the elderly (below 45%) and further decreases with an increasing number of medications administered [59]. This problem is exacerbated in patients with dementia. According to a study conducted in the Polish population, nearly 60% of patients forget to take their medications. Among them, 70% of patients omitted the medication dose that had been forgotten, and 29% declared that they took such a dose as soon as they remembered. It may potentially lead to reduced levels of the active substance, which in turn reduces treatment efficiency or leads to adverse reactions when the level of the active substance increases beyond the safety threshold [57]. The complexity of the treatment regimen and introduction of non-pharmacological guidelines may also pose a problem. Geriatric patients often do not abide by physicians’ guidelines due to an overly complex treatment regimen, prolonged treatment duration, or the introduction of constant changes that are difficult to memorize for the elderly. Furthermore, patients are required to change their previous lifestyles and to introduce recommended modifications such as a limited intake of salt and fluids, tobacco smoking cessation, reduced alcohol consumption, performing physical exercises of moderate activity, and daily body weight monitoring [60]. Those non-pharmacological recommendations are usually negatively received by patients as difficult to introduce or hardly significant, which is a challenge, especially in elderly patients who have been accustomed to their own lifestyle for many years. However, it should be underlined that social support and loneliness are often negative predictors of compliance, especially among older patients. Studies report that living alone was an independent determinant of follow-up appointment-keeping, medication-taking, lifestyle changes, and overall compliance scores. Cognate social support provided by family members and other informal caregivers in the care of HF patients have a positive effect on adherence [61].

## 6. Neurohormones in HF

### 6.1. Natriuretic Peptides

Neurohormones play a major role in cardiology, primarily due to their role in diagnosis and prognosis of various cardiovascular conditions, including acute coronary syndromes and CHF. Among neurohormones, both natriuretic peptides, including N-terminal pro-brain natriuretic peptide (NT-proBNP) and brain natriuretic peptide (BNP), have become established diagnostic and prognostic markers [62]. Measurement of plasma concentrations of natriuretic peptides is recommended as a diagnostic test in patients with symptoms indicative of HF to rule out the diagnosis [1]. However, levels of natriuretic peptides tend to increase with age and may be labile in the presence of comorbidities such as obesity, kidney disease, thyroid disease, or atrial fibrillation [3]. Therefore, the usefulness of natriuretic peptides in elderly patients may be limited. The BED (BNP Usefulness in Elderly Dyspnoeic Patients) study reported that brain natriuretic peptide did not discriminate adequately between cardiac and respiratory origin of acute dyspnea in patients ≥ 60 years [63]. In a meta-analysis of brain natriuretic peptide -guided therapy in patients with HF, where two cohorts divided by age were investigated and compared, similar correlation was observed. A study reported that in patients under 75 years old, therapy guided by NT-proBNP decreased mortality and hospitalization rates; there was no improvement in patients over 75 years of age [64]. While data from the TIME-CHF (Trial of Intensified versus Standard Medical Therapy in Elderly Patients with Congestive Heart Failure) study showed that NT-proBNP-guided therapy is safe in elderly and highly co-morbid HF patients [65].

### 6.2. Copeptine in Elderly Patients

Neurohormones of the arginine vasopressin (AVP) system, including AVP and its surrogate marker copeptin, have also been investigated in elderly patients with HF as potentially useful in clinical practice. AVP is a conventional neurohormone released from the hypothalamus in response to hypovolemia and increased plasma osmolarity. High osmolarity is partially responsible for the increase in AVP concentration in patients with CHF. AVP release in HF is primarily triggered by reduced cardiac output, which leads to insufficient filling of arteries that activates carotid sinus and aortic baroreceptors. Hyponatremia, which is a common occurrence in patients with HF, might further stimulate the release of AVP. Concentration of AVP can also be increased due to other factors, such as angiotensin II [66].

Copeptin, the C-terminal portion of provasopressin, is a novel neurohormone of the AVP system co-secreted with AVP from the hypothalamus; it reflects vasopressin concentration in human plasma and serum. Because of its equimolar secretion with AVP and greater stability, it has been consistently replacing AVP in the clinical setting [47], and accordingly, copeptin has been focused on in studies investigating the co-relation between the AVP system and various clinical conditions [67]. A previous study showed that copeptin was an independent predictor of mortality and rehospitalization in patients with advanced HF [68]. Similar findings were reported in a study on elderly patients, showing an association between copeptin levels and mortality [69].

Combining copeptin with other biomarkers may enhance prognostic capabilities in older patients. A study on 470 elderly patients exhibiting symptoms of HF examined the association between plasma concentrations of copeptin, in combination with concentrations of the NT-proBNP, and mortality. The study found that in this age group elevated copeptin levels, as well as the combination of increased copeptin and NT-proBNP levels, were correlated with the increase in all-cause mortality risk during a median period of 13 years [69]. Similarly, among 172 patients suffering from chronic HF, copeptin and high-sensitivity cardiac troponin T were highly effective in predicting outcomes during long-term follow-up [70].

### 6.3. Other Biomarkers

New biomarkers are under evaluation for their potential to advance the management of patients with HF, but only a few have shown their usefulness for elderly patients.

Suppression of tumorigenicity 2 (ST2) is a member of the interleukin 1 receptor family found in 2 main isoforms: transmembrane or cellular (ST2L) and soluble or circulating (sST2) forms. ST2 is a marker associated with inflammatory disease and fibrosis, and its concentration was found to not be affected by age, renal function, or body mass index [71]. ST2L is a membrane-bound receptor with IL-33 as its functional ligand. IL-33/ST2L signaling triggers the transcription of inflammatory genes, leading to the production of inflammatory cytokines and chemokines and the activation of the immune response. Depending on the circumstances, IL-33 can exhibit a pro- or anti-inflammatory cytokine effect. IL-33 has also been demonstrated to have cardioprotective effects. sST2 is released into the circulation, where it acts as a decoy receptor for IL-33, suppressing the effects of IL-33/ST2L signaling. Thus, elevated circulating sST2 levels diminish the systemic biological effects of IL-33 [72]. ST2 demonstrates predictive value for 30-day and 1-year outcomes in comorbid frail elderly patients with HFpEF in a study comparing four different markers. This study evaluated the prognostic significance of early post-discharge circulating levels of ST2, NT-proBNP, cancer antigen 125 (CA125), and high-sensitivity cardiac troponin T (hs-cTnT). ST2 demonstrated superior predictive capability compared to NT-proBNP for risk prediction of the composite primary endpoint, comprising all-cause mortality and heart HF-related rehospitalization. Thus, establishing its role as a predictive marker in high-risk HFpEF [71].

Another marker that could be useful in the elderly is hs-cTnT; as mentioned earlier, its combined use with copeptin was an excellent prognostic marker for patients with stable HF. It is a marker reflecting myocardial injury, associated with various cardiovascular conditions, including heart failure and myocardial infarction. Plasma concentration of hs-cTnT is routinely checked in patients with HF, particularly in acute HF syndromes. Measurement of cardiac troponin I (cTnI) or cTnT is recommended to identify type 1 myocardial infarction (MI) or acute HF-related injury [72]. However, sensitive troponin levels have also been linked to prognosis in patients with CHF. Longitudinal changes in cTnT concentrations assessed using hs-cTnT have been shown to predict cardiovascular events in these patients. In elderly people, hs-cTn has also shown its usefulness. In the CORONA (Controlled Rosuvastatin Multinational Trial in Heart Failure) trial, elevated hs-cTnT levels in elderly patients with chronic HF of ischemic cause provided independent prognostic and discriminating information beyond that of established risk markers [73].

Although new biomarkers such as growth differentiation factor 15, soluble urokinase-type plasminogen activator receptor, and fibroblast growth factor-23 were observed to have clinical significance in patients with HF and their pathophysiology shows correlation with aging, their usefulness in the elderly population is disputable [74,75,76]. Currently, the data regarding patients over 65 years of age is scarce, and the results are nonconclusive. To properly establish their role in the elderly, further research is needed.

## 7. Conclusions

HF in the elderly is a nuanced and varied problem in clinical practice. The clinical profiles of elderly patients differ from those of younger ones, and non-specific symptoms of HF in this group of patients are associated with a later diagnosis and consequently significantly worse prognosis. To improve the prognosis, a holistic approach is recommended. Addressing the issue of polypharmacy, undertreatment, specific and frequent aging particularities such as frailty and other geriatric conditions, and proper management of comorbidities are vital steps in HF treatment in this group. Special care should be applied to pharmacotherapy to ensure effective and safe treatment for elderly patients. Natriuretic peptides commonly used in HF have limited clinical utility in elderly patients. Other biomarkers, including copeptin, ST2, and high-sensitive troponin, may be useful prognostic tools in this population.
